# Sensitization of Non-Small Cell Lung Cancer Cells to Gefitinib and Reversal of Epithelial–Mesenchymal Transition by Aloe-Emodin *Via* PI3K/Akt/TWIS1 Signal Blockage

**DOI:** 10.3389/fonc.2022.908031

**Published:** 2022-05-23

**Authors:** Minghui Peng, Zhuifeng Zheng, Shaoyang Chen, Le Fang, Rongxiu Feng, Lijun Zhang, Qingnan Tang, Xuewen Liu

**Affiliations:** ^1^ Department of Oncology, Third Xiangya Hospital, Central South University, Changsha, China; ^2^ Department of Radiation Oncology, Hunan Cancer Hospital and the Affiliated Cancer Hospital of Xiangya School of Medicine, Changsha, China; ^3^ Department of Oncology, Loudi Central Hospital, Loudi, China; ^4^ Department of Radiation Oncology, Xiangtan Central Hospital, Changde, China; ^5^ Department of Oncology, Huaihua First People’s Hospital, Changde, China

**Keywords:** aloe-emodin, gefitinib, NSCLC, drug resistance, EMT

## Abstract

**Objective:**

To explore the impacts of AE (aloe-emodin) in gefitinib-resistant NSCLC (non-small cell lung cancer) cells and the corresponding mechanism.

**Methods:**

PC9 and PC9-GR cells were cultured and treated by gefitinib, AE, or the combination of the two drugs. Then, viability, apoptosis, migration and invasion of cells were investigated using CCK-8, TUNEL, wound healing assay, and transwell assay, respectively. Female BALB/c nude mice were employed for the establishment of xenograft tumor models to examine the role of AE in tumor growth.

**Results:**

PC9-GR cells showed reduced apoptosis and enhanced cell viability, migration and invasion upon treatment by gefitinib, compared with PC9 cells. E-cahherin in PC9-GR cells was down-regulated, while Vimentin, Snail2 (or Slug) and Twist1 in PC9-GR cells were up-regulated, compared with PC9 cells. Meanwhile, treatment by a combination of gefitinib and AE significantly strengthened apoptosis of PC9-GR cells, while attenuated their migration and invasion, compared with the control group or treatment by gefitinib or AE alone. WB results showed that AE could reverse EMT and activation of PI3K/AKT signalling pathway in PC9-GR cells. *In vivo* experiments showed that tumor growth and EMT of PC9-GR cells were dramatically repressed after treatment by a combination of AE and gefitinib. Additionally, the use of SC97 (a PI3K/Akt pathway activator) could counteract the effects of AE in gefitinib-resistant PC9 cells.

**Conclusions:**

AE could enhance the gefitinib sensitivity of PC9-GR cells and reverse EMT by blocking PI3K/Akt/TWIS1 signal pathway.

## Introduction

Although great advances in cancer treatment have been achieved in recent decades, the prognosis of patients remains unsatisfactory due to the development of drug resistance during the treatment (1, 2). Lung cancer, which consists of about 85% NSCLC and 15% SCLC, is the top cancer globally and a leading cause of cancer death (3, 4). The therapeutic techniques for NSCLC, which depend on pathological types, clinical stages as well as other medical conditions, include surgery, adjuvant chemotherapy, immunotherapy, and targeted therapy (5).

Epidermal growth factor tyrosine inhibitors (EGFR-TKIs) are considered typical paradigms of targeted therapies in NSCLC, but acquired drug resistance unavoidably limits the clinical efficacy of EGFR-TKIs, which therefore makes fighting against EGFR-TKI resistance an absorbing topic for NSCLC treatment (6, 7). Gefitinib, one of the widely used EGFR-TKIs, has been approved for the first-line treatment of NSCLC with sensitizing EGFR mutations (exon 19 deletion or L858R point mutation) (8), while drug resistance in cancer cells contributes to the limited clinical efficacy of the targeted drug. Therefore, safe yet effective antitumor drugs or treatments are urgently needed for the management of LC.

The intimate connection between epithelial-mesenchymal transition (EMT) activation and drug resistance are founded in many diseases, including NSCLC (9). In addition, several mechanisms including abnormal activation of PI3K/Akt pathway have been proved to generate drug resistance in cancer (10). One study reported that miR-30a-5p overexpression can overcome EGFR-TKI resistance in NSCLC by regulating PI3K/Akt pathway (11). Fang et al. revealed the distribution of common mutations involving PI3K activation in EGFR-TKI resistant patients, which were difficult to overcome (12). Zhou et al. found that PI3K/Akt signaling pathway was suppressed by miR-200c to strengthen the sensitivity of drug-resistant NSCLC cells to gefitinib (13). Hao et al. demonstrated that IRS4 conferred therapy resistance in lung cancer cells through activating PI3K/Akt signaling (14). These studies collectively indicate PI3K/Akt signaling pathway may be a promising target of overcoming the gefitinib resistance in NSCLC.

As a natural anthraquinone derivative and an active ingredient of Chinese herbs, Aloe-emodin (AE, **Figure 2A**) exhibits pharmacological effects on various diseases, including liver fibrosis, LC, and leukemia (15). Ma et al. found that AE could trigger EMT in SkBr3 cells (breast cancer cells) to repress cancer metastasis (16). Dou et al. reported that AE could alleviate renal fibrosis by repressing PI3K/Akt/mTOR signaling pathway both *in vivo* and *in vitro* (17). Additionally, it has been demonstrated that AE could mitigate drug resistance of breast cancer cells and melanoma cells, indicating a regulatory role of AE in drug resistance (18, 19). Nonetheless, effects of AE on drug resistance of NSCLCs have not been fully explored.

In this study, the role of AE in the development of drug resistance of NSCLCs and its underlying mechanism were investigated to provide insights for NSCLC treatment.

## Material and Methods

### Cell Culture

The PC9 cell line was obtained from the Chinese Academy of Science (Shanghai, China) and cultured in RPMI 1640 with 10% FBS under 5% CO_2_ at 37°C. Then, gefitinib was added with elevating concentrations to develop and maintain the resistant cell line for half a year (20).

PC9-GR and PC9 cells were then subjected to gefitinib of different concentrations (0, 0.01, 0.1, 1, 10, 100 μM), while PC9-GR cells were further treated by DMSO (the control group), 10 μM gefitinib (the gefitinib group), 10 μM AE (the AE group), the combination of gefitinib and AE (the gefitinib+AE group), and the combination of gefitinib and AE plus SC97 (the gefitinib+AE+SC97 group).

### Cell Viability

Cell viability was determined using CCK8. Briefly, cell seeding was executed in 96-well plates and a confluence was reached in 24 h. Then, cells were treated with gefitinib of different concentrations and/or AE. After 24 h, the cells were further incubated with CCK‐8 for 120 min, and the viable cells were subsequently counted according to the absorbance at 450 nm.

### Cell Migration

Cell migration was detected using the wound healing assay. First, cell seeding was executed in 6‐well plates and cultured for 24 h. Thereafter, a single scratch was created using a 100 μL pipette tip, and the cells were incubated with FBS‐free culture medium containing DMSO or gefitinib and/or AE after being rinsed with PBS. Finally, the scratches at 0 and 24 hours were captured using an Olympus IX71 inverted microscope.

### Cell Invasion

Cell invasion was assessed using the transwell assay. 60 μL of Matrigel was added into each chamber, and the cells were seeded in the upper chamber of the insert with or without drugs. Then, the upper chamber was cleaned and cells on the membrane bottom were fixed and stained after a 24-hour incubation. A light microscope was used to count the cells on the membrane bottom.

### Cell Apoptosis

Cell apoptosis was assessed using the TUNEL assay. First, cells were rinsed with PBS twice, incubated with formaldehyde for 20 min, and permeabilized with 0.1% TritonX‐100 for 10 min. Then, cells were incubated in PBS for 5 min, TdT reaction mixture was added and cells were incubated in a dark humidified chamber at 37°C for 60 min. Thereafter, 2 × SSC buffer was used to stop the reaction, and DAPI (Abcam, MA, USA) was used to counterstain the cell nuclei after being rinsed with PBS. Finally, a fluorescence microscope was used to capture images.

### qPCR

Total RNA extraction from PC9-GR cells was executed using Trizol Reagent. Then, cDNA of the mRNA was obtained, and the mixture of cDNA and SYBR Green was subjected to qPCR by a real-time PCR System. The results were quantified using the 2^-ΔΔCT^ method. GAPDH was employed as an internal reference for mRNA standardization in each sample. Specific primers used for qPCR are listed in **Table S1**.

### Western Blot

PC9-GR or PC9 cells were lysed in RIPA buffer to extract total proteins, which were subsequently segregated on SDS‐PAGE gels and transferred to PVDF membranes. Then, primary antibodies were added and cells were incubated at 4°C overnight upon blockage of 5% skim milk. After three rinsing cycles, appropriate secondary antibodies were added and cells were incubated at 37°C for 2 h. Finally, samples were characterized by the ECL system and quantified by Image J. The detailed information of antibodies used is listed in **Table S2**.

### *In Vivo* Experiments

Female BALB/c nude mice (age = 4-5 weeks and weight = 16-20 g) were provided by the Guangdong Medical Laboratory Center (China). The animals were raised in a SPF environment, which were given *ab libtum* to water and standard chow. After acclimated for one week, subcutaneous inoculation of 1 × 10^7^ PC9-GR cells was executed into the right flank of the animals (*n* = 4 in each group) to develop xenograft models. Once the tumor volume approached 50 mm^3^ by average, gefitinib (30 mg/kg), AE (30 mg/kg) and their combination were administered, respectively, while the same volume of saline was administered as the control group. The tumor size was evaluated every week using a digital caliper, while tumor volumes were calculated by: Volume = (Length × Width^2^)/2. The mice were euthanized on Day 21 and tumors were weighed immediately.

### Statistical Analysis

Student’s t-tests were employed to compare the two groups. One- or two-way ANOVA was performed on multiple-group comparison. All data were in the format of mean ± standard deviation (SD) and the three experiments were independent from each other. P<0.05 indicated statistically significant differences.

## Results

### Gefitinib-Resistance of PC9-GR Cells is Related to EMT

**Figure 1:**
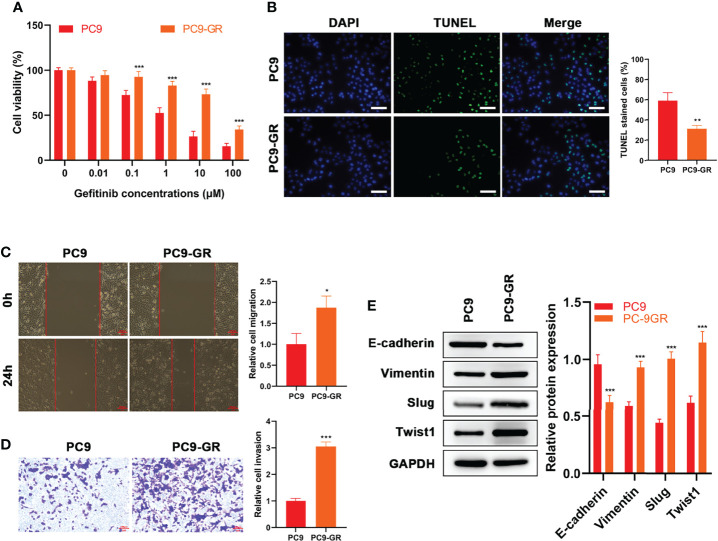
EMT Occurred in Gefitinib‐Resistant Cells. **(A)** CCK8 results of PC9 and PC9-GR cells at different concentrations of gefitinib treatment **(B)** TUNEL results, **(C)** wound healing assay results and **(D)** Transwell assay of PC9 and PC9-GR cells at 1 μM of gefitinib **(E)** Expressions of EMT-related proteins (E-cadherin, Vimentin, Slug and Twist1) in the two cell lines at 1 μM of gefitinib detected by WB results. Scale bar = 100 μm. *p<0.05, **p<0.01, ***p<0.001.

First, gefitinib‐resistant PC9 cells were constructed and their viability in gefitinib was enhanced compared with their parent PC9 cells (p < 0.001, **Figure 1A**). Then, the effects of 1 μM gefitinib on apoptosis, migration, and invasion of the cell samples were examined. Investigation by TUNEL assay showed that the apoptosis rate of PC9-GR cells was prominently lower than that of PC9 cells (p = 0.005, **Figure 1B**). Enhanced migration (p = 0.016, **Figure 1C**) and reduced apoptosis (p < 0.001, **Figure 1D**) were founded in PC9-GR cells, as shown in the results of wound healing assay and cell invasion assay. WB results indicated that the E-cadherin level was significantly higher in PC9 cells than in PC9-GR cells, while levels of Vimentin, Slug, Twist1 in PC9 cells were significantly lower in PC9 cells than in PC9-GR cells (p < 0.001, **Figure 1E**). In summary, EMT was observed in gefitinib‐resistant cells and it might be associated with the development of gefitinib resistance.

### AE Weakens the Gefitinib Resistance of PC9-GR Cells

**Figure 2:**
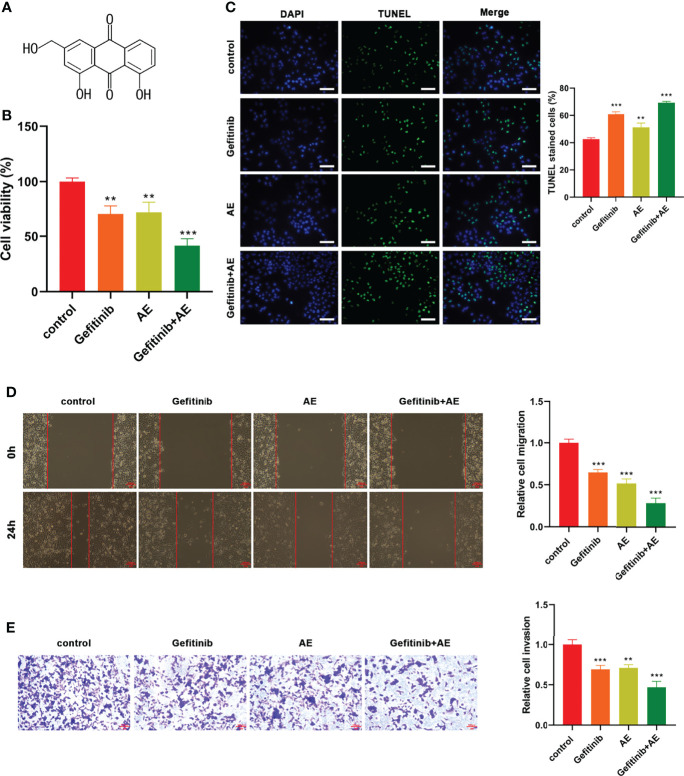
AE Could Enhance the Gefitinib Sensitivity of PC9-GR Cells. **(A)** Chemical structure of AE. **(B)** CCK8 results, **(C)** TUNEL assay results, **(D)** wound healing assay results, and **(E)** Transwell assay results of PC9-GR cells after different treatments. Scale bar = 100 μm. **p<0.01, ***p<0.001.

The effects of AE (**Figure 2A**) on PC9-GR cells were explored. The results showed that the presence of AE led to a significant decrease in cell viability (p = 0.005, **Figure 2B**), migration (p < 0.001, **Figure 2D**), and invasion (p = 0.001, **Figure 2E**), and an increase in apoptosis (p = 0.003, **Figure 2C**). Meanwhile, the combination of AE and gefitinib achieved a notable suppression on the proliferation, migratory and invasive abilities of PC9-GR cells, which was refractory to gefitinib treatment alone (p < 0.001, **Figures 2B–E**). These results demonstrated that the combination was more effective than either one alone, and AE could reduce the resistance of PC9-GR cells to gefitinib.

### AE Reverses EMT in PC9-GR Cells

**Figure 3:**
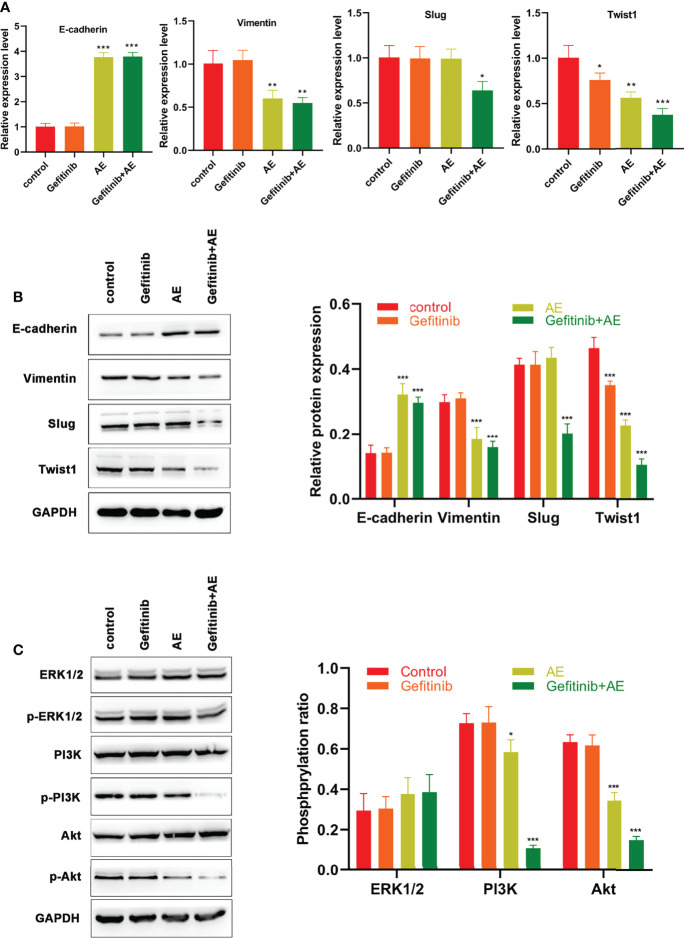
AE Could Reverse EMT Induced by Drug Resistance in PC9-GR Cells. **(A)** qPCR results of E-cadherin, Vimentin, Slug and Twist1 for PC9-GR cells. **(B)** WB results of E-cadherin, Vimentin, Slug, and Twist 1 for PC9-GR cells **(C)** WB results of the PI3K/Akt pathway related proteins for PC9-GR cells. *p<0.05, **p<0.01, ***p<0.001.

Previous studies have demonstrated the presence of EMT during the development of drug resistance (21–23). Hence, effects of AE on EMT in PC9-GR cells were investigated. qPCR analysis revealed that the E-cadherin levels increased significantly when treated with AE alone (p < 0.001), whereas the levels of Vimentin (p = 0.009) and Twist1 (p = 0.002) decreased under the administration of AE alone (**Figure 3A**). The combination of AE and gefitinib exhibited a stronger effect on reversing EMT, as evidenced by a significant increase in E-cadherin levels (p < 0.001) and a decrease in levels of Vimentin (p = 0.004), Slug (p = 0.022) and Twist1 (p < 0.001) (**Figure 3A**). These results were further corroborated by WB analysis (**Figure 3B**). Meanwhile, the MAPK/ERK and PI3K/Akt signaling pathways were detected in PC9-GR cells with the treatment of AE. There is no significant difference in the phosphorylation of ERK1/2 among all groups (all p > 0.05), while a remarkable decline in the phosphorylation of PI3K (p =0.035) and Akt (p < 0.001) was conformed upon AE treatment (**Figure 3C**). Notably, the combination of AE and gefitinib exhibited a stronger suppression effect than AE alone on PI3K and Akt phosphorylation (all p< 0.001, **Figure 3C**). These results indicated that AE could reverse the EMT in PC9-GR cells, and inactivate the PI3K/Akt pathway.

### AE Enhances the Gefitinib Sensitivity of Xenograft Tumors and Reverses the EMT *In Vivo*

**Figure 4:**
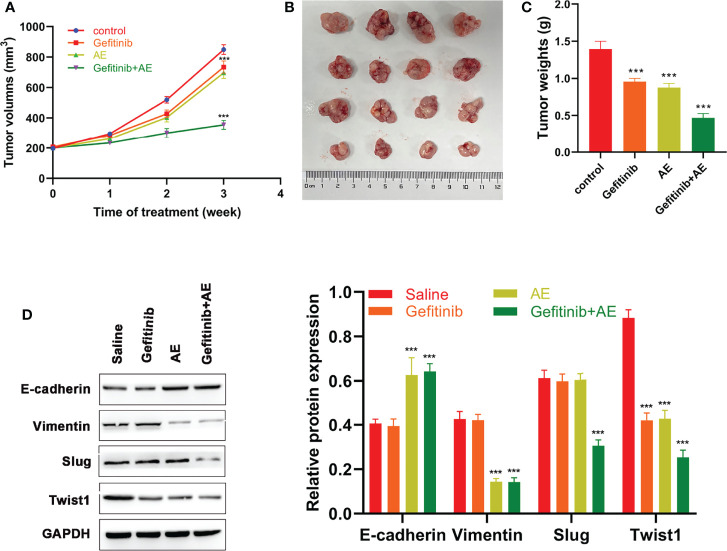
AE Could Enhance the Sensitivity of Xenograft Tumors to Gefitinib. **(A)** Volumes of xenograft tumors, **(B)** tumor growth, **(C)** weights of xenograft tumors and **(D)** WB results of EMT-related proteins of different groups after different treatments. ***p<0.001.

To further verify the conclusions, *in vivo* experiments were conducted. Compared with the control group, gefitinib alone showed a slight repression in tumor growth, while the combination of AE and gefitinib achieved a notable growth suppression on PC9-GR xenografts (all p < 0.001, **Figures 4A–C**). Similar to the *in vitro* results, WB results suggested that E-cadherin was significantly up-regulated when treated with AE and gefitinib, whereas Vimentin, Slug and Twist1 were down-regulated when treated with the combination of these two drugs (all p < 0.001, **Figure 4D**). In summary, AE could enhance the sensitivity of xenograft tumors to gefitinib and reverse the EMT *in vivo*.

### AE Strengthens Gefitinib Sensitivity of PC9-GR Cells and Reverses EMT *Via* PI3K/Akt Pathway

**Figure 5:**
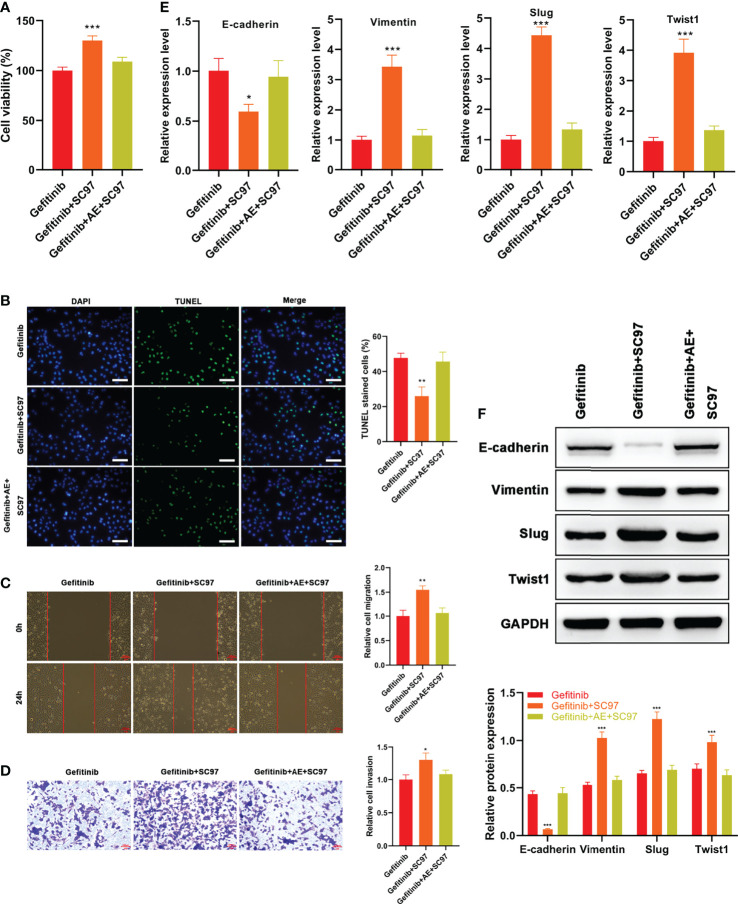
AE Strengthened the Gefitinib Sensitivity of PC9-GR Cells by Reversing EMT *Via* PI3K/Akt/TWIS1 Pathway. **(A)** Relative cell viability of PC9-GR cells treated with different treatments **(B)** TUNEL assay results, **(C)** wound healing assay results, **(D)** Transwell assay results, **(E)** qPCR results of EMT-related genes, and **(F)** WB results of EMT-related genes in PC9-GR cells after different treatments. Scale bar = 100 μm. *p<0.05, **p<0.01, ***p<0.001.

Based on the results mentioned above, AE was supposed to reverse EMT and block PI3K/Akt pathway. Therefore, a PI3K activator SC97 was used to verify this hypothesis. PC9-GR cells were subjected to gefitinib, SC97, or the combination of gefitinib and AE plus SC97. Then, viability, apoptosis, migration, and invasion of the cells were determined. Consistent with the foregoing results, administration of SC97 led to attenuated apoptosis (p = 0.003, **Figure 5B**), and enhanced viability (p < 0.001, **Figure 5A**), migration (p = 0.002, **Figure 5C**) and invasion (p = 0.012, **Figure 5D**) of PC9-GR cells, while AE significantly alleviated this effect (all p > 0.05, **Figures 5A–D**).

qPCR and WB results suggested AE reversed the reduced expression of E-cadherin and enhanced expressions of Vimentin, Slug and Twist1 by SC97 (all p > 0.05, **Figures 5E, F**). Overall, the results revealed that AE could strengthen the gefitinib sensitivity of PC9-GR cells and reverse EMT by blocking PI3K/Akt/TWIS1 pathway.

## Discussion

Many advances in therapeutic strategies for cancer have been made in recent years, but the occurrence of drug resistance cuts down the curative effects of therapies (24, 25). Among the therapeutic methods for NSCLC, targeted therapy is characterized by high efficiency, reduced side effects, and wide adaptability (26). Gefitinib is a commonly used targeted drug for the treatment of NSCLC with favorable efficacy, but the drug resistance gradually weakens the clinical therapeutic effect of the targeted drug, resulting in an ineffective prognosis. Owing to a long period for development and clinical applications of new drugs, the treatment of many patients might not be timely. Therefore, exploration of a potential adjuvant to gefitinib in NSCLC treatment to overcome drug resistance is promising in improving the treatment efficacy for patients.

The anticancer properties of AE on several types of cancers, including bladder cancer, cervical cancer, and colon cancer, have been explored (27). The results of this study confirmed the anticancer properties of AE on NSCLC by treating PC9-GR cells or xenograft models with a combination of gefitinib and AE or AE alone. Cheng et al. presented that AE sensitized tumor cells to chemotherapeutic agents, indicating the potential of AE for improving drug resistance (18). In this study, AE could improve the drug resistance of PC9-GR cells and xenograft models.

It has been demonstrated that EMT plays an important role in drug resistance in various cases, such as lung cancer and breast cancer, etc. (22, 28, 29). Likewise, the results of this study showed that E-cadherin was significantly down-regulated in PC9-GR cells, whereas vimentin, slug, and twist1 were up-regulated in PC9-GR cells, indicating the presence of EMT in drug-resistant cells. Additionally, the combination of AE and gefitinib or AE alone can reverse the EMT both *in vitro* and *in vivo*.

It has been found that FAT4 (FAT tumor suppressor homolog 4) could regulate the autophagy and the EMT process in colorectal cancer cells by blocking the PI3K-Akt signaling pathway (30). Meanwhile, GPER1 regulates the EMT process by blocking the PI3K-Akt signaling pathway in gastric cancer (31). In addition, the ERK1/2 signal pathway was involved in EMT (32–36). These studies suggested that the ERK1/2 or PI3K/Akt signaling pathway might be involved in drug resistance.

The determination of phosphorylation of relevant indices of ERK1/2 and PI3K-Akt pathways in this study showed no significant difference in the activation of ERK signaling pathway, while the activation of PI3K/Akt pathway exhibited significant upregulation in the AE group and the gefitinib+AE group, suggesting that the PI3K/Akt signaling pathway participated the AE regulation in drug resistance. Additionally, SC97, a PI3K/Akt pathway activator, was employed to verify that AE strengthened the gefitinib sensitivity of PC9-GR cells and reversed EMT *via* the PI3K/Akt axis.

To sum up, AE can induce the apoptosis and reduce the viability, migration and invasion of gefitinib-resistant NSCLC cells. More importantly AE enhance the gefitinib sensitivity of PC9-GR cells in NSCLC cells and xenograft models and reverse EMT by blocking PI3K/Akt signaling pathway. These findings highlight the anti-resistance property of AE, indicating that AE is a promising sensitizer of gefitinib in NSCLC treatment. Also, this study provides insights for other researchers to combat drug resistance, and offers a novel question that whether AE could be used as an adjuvant in chemotherapy and targeted therapy for other diseases.

## Data Availability Statement

The original contributions presented in the study are included in the article/**Supplementary Material**. Further inquiries can be directed to the corresponding author.

## Author Contributions

Conceptualization, MP, ZZ, SC and XL. Validation and formal analysis, LF and RF. Investigation anddata curation, LZ and QT. Writing—original draft preparation, MP, ZZ and SC. Writing—review and editing, XL. All authors have read and agreed to the published version of the manuscript.

## Conflict of Interest

The authors declare that the research was conducted in the absence of any commercial or financial relationships that could be construed as a potential conflict of interest.

## Publisher’s Note

All claims expressed in this article are solely those of the authors and do not necessarily represent those of their affiliated organizations, or those of the publisher, the editors and the reviewers. Any product that may be evaluated in this article, or claim that may be made by its manufacturer, is not guaranteed or endorsed by the publisher.
