# Supplementary material for: Sensitization of Non-Small Cell Lung Cancer Cells to Gefitinib and Reversal of Epithelial–Mesenchymal Transition by Aloe-Emodin Via PI3K/Akt/TWIS1 Signal Blockage
Source: Front Oncol. 2022 May 23;12:908031. doi: 10.3389/fonc.2022.908031 (PMC9168594; doi:10.3389/fonc.2022.908031)
Supplement: Supplementary file 1 [file Table_1.docx]

**Table S1- primer Sequences**

| **Gene** | **Sequence** | |
| --- | --- | --- |
| E-cadherin | Forward (5’-3’) | GCTCTGAGGAGTGGTGCATT |
|  | Reverse (5’-3’) | GCAATTTCTCGGCCCCTTTC |
| Vimentin | Forward (5’-3’) | GGTTCAGGTTTCATTCATGCCT |
|  | Reverse (5’-3’) | TTGCGCTCCTGAAAAACTGC |
| Slug | Forward (5’-3’) | ACGCAAGTATACACACACTGGA |
|  | Reverse (5’-3’) | GAGCACCTATAGCTCCGTGC |
| Twist1 | Forward (5’-3’) | TTTTAAAAGTGCGCCCCACG |
|  | Reverse (5’-3’) | ACAGCCGCAGAGACCTAAAC |
| GAPDH | Forward (5’-3’) | GCAACTAGGATGGTGTGGCT |
|  | Reverse (5’-3’) | TCCCATTCCCCAGCTCTCATA |
