# Supplementary material for: Sensitization of Non-Small Cell Lung Cancer Cells to Gefitinib and Reversal of Epithelial–Mesenchymal Transition by Aloe-Emodin Via PI3K/Akt/TWIS1 Signal Blockage
Source: Front Oncol. 2022 May 23;12:908031. doi: 10.3389/fonc.2022.908031 (PMC9168594; doi:10.3389/fonc.2022.908031)
Supplement: Supplementary file 2 [file Table_2.docx]

**Table S2 The information of antibodies used in Western blot**

| **Antibody** | **Manufacturers** | **Cat.no** |
| --- | --- | --- |
| E-cadherin | Abcam | ab231303 |
| Vimentin | Abcam | ab92547 |
| Slug | Abcam | ab27568 |
| Twist | Abcam | ab50887 |
| ERK1/2 | Abcam | ab17942 |
| PI3K | Abcam | ab191606 |
| Akt | Abcam | ab8805 |
| p-ERK1/2 | Abcam | ab278538 |
| p-PI3K | Abcam | ab278545 |
| p-Akt | Abcam | ab81283 |
| GAPDH | Abcam | ab181602 |
| HRP Anti-Rabbit IgG antibody | Abcam | ab288151 |
| Goat Anti-Mouse IgG H&L (HRP) | Abcam | ab6789 |
